# Therapeutic Drug Monitoring of Isavuconazole: Serum Concentration Variability and Success Rates for Reaching Target in Comparison with Voriconazole

**DOI:** 10.3390/antibiotics10050487

**Published:** 2021-04-23

**Authors:** Malene Risum, Mai-Britt Vestergaard, Ulla Møller Weinreich, Marie Helleberg, Nadja Hawwa Vissing, René Jørgensen

**Affiliations:** 1 Unit of Mycology, Department of Bacteria, Parasites and Fungi, Statens Serum Institut, Artillerivej 5, 2300 Copenhagen, Denmark; mlri@ssi.dk; 2 Department of Haematology, Copenhagen University Hospital, Rigshospitalet, 2100 Copenhagen, Denmark; 3 Department of Respiratory Diseases, Aalborg University Hospital, 9000 Aalborg, Denmark; maives@rn.dk (M.-B.V.); ulw@rn.dk (U.M.W.); 4 The Clinical Institute, Aalborg University, 9000 Aalborg, Denmark; 5 Department of Infectious Diseases, Copenhagen University Hospital, Rigshospitalet, 2100 Copenhagen, Denmark; Marie.Helleberg@regionh.dk; 6 Department of Paediatrics and Adolescent Medicine, Copenhagen University Hospital, Rigshospitalet, 2100 Copenhagen, Denmark; nadja.hawwa.vissing@regionh.dk

**Keywords:** therapeutic drug monitoring, antifungal drug, aspergillosis, mucormycosis

## Abstract

Isavuconazole (ISZ) is used in the treatment of aspergillosis and mucormycosis. The purpose of this study was to evaluate the therapeutic drug monitoring (TDM) of ISZ samples from a clinical setting performed at Statens Serum Institut. **Materials/methods:** Isavuconazole serum concentrations were determined by fluorescent detection on a UHPLC. Serum-ISZ (s-ISZ) results were included and compared to those of serum-voriconazole (s-VRZ) in a 33 month period from March 2017. Clinical data were obtained for patients receiving ISZ. The therapeutic range was initially 2–10 mg/L, but was adjusted to 2–5 mg/L during the study period except for selected patients with Mucorales infections who received off-label doses of ISZ. **Results:** A total of 273 s-ISZ and 1242 s-VRZ measurements from 35 and 283 patients, respectively, were included. Seventeen patients had received both ISZ and VRZ with TDM within the study period. The median s-ISZ was 4.3 mg/L (0.5–15.4 mg/L) with 83% of measurements within the therapeutic index. The median s-VRZ was 2.6 mg/L (0.2–21.9 mg/L) with 67% of measurements within the therapeutic index. The median intra-/interindividual coefficient of variation (CV) was 43.4%/54.8% for ISZ compared to 53.2%/83.3% for VRZ. For patients receiving ISZ, the adverse events were mostly gastroenteric and few drug–drug interactions were observed. Furthermore, immediate change from ISZ to VRZ treatment seemed to lead to prolonged metabolism of ISZ with detection up to 35 days after discontinuation. **Conclusions:** The majority of patients achieved s-ISZ levels well within the therapeutic range with less intra/interindividual CV than patients receiving VRZ.

## 1. Introduction

Isavuconazole (ISZ) is approved for treatment of invasive aspergillosis and mucormycosis. According to current guidelines on mucormycosis, liposomal amphotericin B is strongly recommended as the first line therapy. However, in the case of preexisting renal compromise, ISZ and posaconazole infusion can be used [1]. Like other azole-based antifungals, ISZ is highly protein bound, but is distinct by having a long terminal half-life of 56–104 h [2]. It is metabolized via CYP3A4 and CYP3A5 and is a moderate inhibitor of CYP3A4 [3]. Well-known side effects of ISZ are gastroenteric and hepatic, but generally it is considered well tolerated [3]. Isavuconazole has been found to be non-inferior to voriconazole (VRZ) in the treatment of invasive aspergillosis and to have an effect in patients with mucormycosis in whom amphotericin B was not tolerated or was inappropriate [4,5]. On the contrary, ISZ did not show non-inferiority to caspofungin in the treatment of candidaemia and candidiasis [6]. In addition to treatment, ISZ was found to be safe as prophylaxis in a clinical trial with acute myeloid leukemia and myelodysplastic syndrome patients of which almost half received targeted treatments such as venetoclax and FLT3 inhibitors [7]. Isavuconazole as prophylaxis has also been reported in other studies [8,9,10,11] as well as the use of ISZ in children [12] and patients with chronic pulmonary aspergillosis (CPA) [13,14]. Although ISZ has been used as prophylaxis, this is a non-approved and off-label intervention. There are no randomized clinical trials that compare its use as prophylaxis to posaconazole and VRZ.

The pharmacokinetic features of ISZ identified during clinical development have not provided a clear need for therapeutic drug monitoring (TDM) [15,16]. Nevertheless, a recent study based on 19 patients receiving ISZ recommended a therapeutic range between 2 and 5 mg/L based on reported adverse events, which led to discontinuation in 16% (3/19) of patients [17]. We monitored the ISZ serum concentrations in 35 patients during a period of 33 months and present the results in comparison to VRZ serum concentration measurements in 283 patients receiving VRZ. We also present clinical data for patients who received ISZ.

## 2. Materials & Methods

### 2.1. Study Design

This was a retrospective study including trough samples received at Statens Serum Institut (SSI) from 1 March 2017 to 1 December 2019.

### 2.2. Clinical Data and Definitions

Clinical data were obtained for patients in Denmark who had received ISZ and who had TDM performed at SSI during the study period. One patient was switched from ISZ to VRZ, but only had s-VRZ performed at SSI and was therefore also included in the clinical data, but not in the TDM values for ISZ. TDM values for that one patient were performed at another laboratory using a LC-MS-based method.

Body weight, information on underlying disease, indication, co-morbidity and treatment for other diseases were retrieved. Dosages, dose adjustments and duration of ISZ treatment within the study period were extracted when stated in the chart. Dose adjustments were performed when either a TDM value was below or above the therapeutic range or after mycological consultation according to species. Information on adverse events was obtained if ascribed to ISZ by the treating physician. Information on all drug–drug interactions was systematically registered when the patient received other drugs also metabolized through CYP3A4 and known to have potential azole interactions. Overall, 42-day mortality was determined from the initiation date of ISZ treatment in order to compare it to other studies.

The SSI initially recommended a therapeutic index for ISZ between 2–10 mg/L, but adjusted the range to 2–5 mg/L in mid-August 2019 in accordance with the study by Furfaro et al. [17]. In mucormycosis a trough level well above the individual MIC was preferred until fungal control was achieved in the reported patients. The recommended therapeutic index for VRZ at our institution was 1–5.5 mg/L throughout the study period [18]. However, a trough level of 2–5.5 mg/L is recommended for invasive *Aspergillus flavus* and *Aspergillus niger*. Data analysis for s-ISZ and s-VRZ was performed under the assumption that a result below detection level (0.2 mg/L) was equal to no drug intake and a mistaken requisition. The successful target rate was defined as the rate of serum levels within the stated therapeutic range relative to the total number of measurements. To express the precision of the measured patient serum concentrations, coefficient of variations (CV) were determined for both ISZ and VRZ.

The study was approved by the local institution (Journal number 20/04568) and clinical data for patients receiving ISZ were retrospectively obtained after permission by the Danish Patient Safety Authority (Journal number 31-1521-36).

### 2.3. Chemicals and Standards

Isavuconazole for calibration standards and control was supplied by Basilea Pharmaceutica Ltd. A 5.000 mg/L ISZ stock solution was prepared in dimethyl sulfoxide (Sigma-Aldrich) and diluted to 160 mg/L in filtered pooled human plasma (BioWest). Isavuconazole calibration standards were prepared in filtered pooled human plasma at concentrations of 0.5, 1.0, 4.0, 8.0 and 16.0 mg/L. A 2.0 mg/L ISZ calibration control was prepared separately. The chemicals for sample preparation and HPLC were obtained from the itraconazole/posaconazole/voriconazole reagent kit for TDM (ChromSystems).

### 2.4. Quantification of Azole Plasma Concentrations

Voriconazole serum concentrations were determined by the reagent kit for TDM from ChromSystems. Isavuconazole serum concentrations were determined by a validated adaptation of the same reagent kit for TDM as previously described [19]. Using this method the reported lower limit of quantification is 0.2 mg/L and within the range of 0.2 to 20.0 mg/L the overall inter- and intraday accuracy and precision are 101.1% and 2.7%, respectively. Briefly, 100 μL of plasma sample was mixed with 25 μL of internal standard and 25 μL of precipitation reagent I. After vortexing, 200 μL of precipitation reagent II was added, and the mixture was vortexed for 30 s before centrifuging for 5 min at 15,000× *g*. The supernatant was transferred to a glass vial and placed in the autosampler followed by injection of 20 μL sample onto the HPLC column (ChromSystems).

### 2.5. Instruments and Chromatographic Conditions

Analyses were carried out on a Vanquish UHPLC system (Thermo Scientific) consisting of a quaternary pump (VF-P20-A), an autosampler with thermostat and column compartment (VH-A10-A and VH-C10-A) and a fluorescence detector (VF-D50-A). The fluorometric detection wavelengths were set to 261 and 366 nm for emission and excitation, respectively. The data were acquired and processed using Chromeleon 7.2 SR4. Chromatographic separation was carried out on an HPLC column with a column temperature of 25 °C and an isocratic flow of 1.2 mL/min for 14 min per sample.

### 2.6. Statistical Methods

CV for all patients (CV interindividual) and CV for each patient (CV intraindividual) for ISZ and VRZ were determined. CV intraindividual was determined when more than one value from the same patient was present. To enable comparison to other studies we stated the CV median intraindividual values.

TDM of ISZ values from some of the patients with Mucorales have previously been published [20]. Some TDM results are published in the abstract book of the European Congress of Clinical Microbiology and Infectious Diseases 2020 [21].

## 3. Results

### 3.1. Clinical Data

Characteristics of the 36 patients receiving ISZ and indications are shown in Table 1. Empirical use was most often seen in hematological patients 38% (6/16). In the pediatric group the indication for switching from VRZ to ISZ was failure to reach target TDM on VRZ or attempts to diminish certain drug–drug interactions, acknowledging the off-label use of ISZ in children.

Ten patients had a proven invasive fungal infection of which five had proven mucormycosis, three had proven invasive aspergillosis, one had another proven mold infection, and one had hyphae shown paranasally, but without species identification. Four patients had a probable and one a possible invasive mold disease. In 21 patients clinical data were either inadequate to further define criteria for probable invasive mold disease (*n* = 5), or the patient lacked a host factor (*n* = 16) to fulfil the revised criteria from the European Organization for Research Treatment of Cancer/Mycoses Study Group (EORTC/MSG) [22] (Table 1). Of the 16 who lacked a host factor, 7 had chronic obstructive pulmonary disorder (COPD).

Twenty-four patients received loading dosages with ISZ according to recommendations. For three patients, information regarding loading was not retrievable, of which one patient had no retrievable data for dosages. Nine patients initiated a maintenance dosage of which eight patients had received prior VRZ or posaconazole or had a low body weight. Patients received ISZ maintenance dosages of 150 mg up to 600 mg per day. The lowest dosage was given in a pediatric patient without loading. All five patients with Mucorales were closely monitored and received loading dosages with ISZ and varying maintenance dosages between 200 to 600 mg per day according to the TDM results, aiming for a target level above 4 mg/L and above 6 mg/L in some of the patients. Dosage and response curves for four of the five Mucorales patients are shown in the Supplementary Material Figure S1. Seven other patients also received intermittently increased dosages of ISZ from 300 to 600 mg a day of which five of them received increased dosages due to low TDMs.

Half (18/36) of patients who received ISZ had also received VRZ within the study period. Forty-two percent of patients (15/36) had received VRZ prior to ISZ. Among those, 60% (9/15) had shifted from VRZ to ISZ due to well-known side effects of VRZ. A total of 3 out of the 18 patients received ISZ first, but were switched to VRZ after the finding of a fungal species for which VRZ was the drug of choice.

### 3.2. Serum Concentrations

#### 3.2.1. Serum Isavuconazole

**Figure 1:**
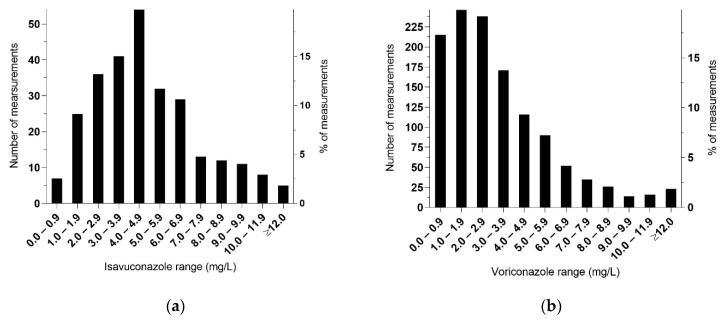
Distribution range of serum concentrations. (**a**) 273 s-ISZ concentrations measured from 35 patients at SSI. Patients received various dosages and the plots do not describe exposures after administration of standard dosing. (**b**) 1242 s-VRZ concentrations measured from 283 patients.

**Figure 2:**
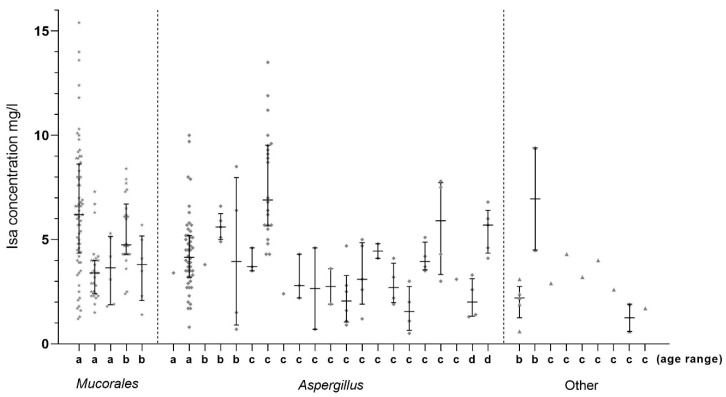
Serum concentration levels of 35 patients at Danish hospitals receiving ISZ with various dosages. The serum concentrations were determined by HPLC with a fluorescence detector during a period of 33 months. The age of the patients at the beginning of treatment is indicated on the *x*-axis and patient groups are separated by a dashed line. a: ≤25 years, b: 26–50 years, c: 51–75 years and d: >75 years. One patient with *Aspergillus* had *Aspergillus flavus* and was treated empirically with ISZ until species identification, then was switched to VRZ.

A total of 280 s-ISZ measurements were performed. Additionally, five measurements were performed outside our laboratory at the time of the ongoing fungal infection in one patient who received VRZ, and who had previously received ISZ. Seven measurements were below a detectable value of <0.2 mg/L, which were in accordance with clinical information on no intake or no adherence. When excluding these seven measurements as well as those performed at another laboratory, 273 measurements of s-ISZ from 35 patients were included in the analysis. The dispersion of TDM levels and number of samples is shown in Figure 1a. The median value was 4.3 mg/L (range: 0.5–15.4 mg/L). Among the five patients with Mucorales, with 127 samples the median value was 4.8 mg/L (1.2–15.4) and among the 126 samples from 20 patients with *Aspergillus* the median value was 4.3 mg/L (range: 0.5–13.5 mg/L). The number of measurements and s-ISZ levels for each individual patient is shown in Figure 2.

The median s-ISZ was 4.3 mg/L among the 12 patients who received increased dosages including the five Mucorales patients, 3.6 mg/L among the seven non-Mucorales patients (specifically) who received intermittently increased dosages and 4.6 mg/L among the 22 patients who only received on-label dosages (Table 2).

When taking the adjusted recommended therapeutic range into account, a total of 227 out of 273 TDMs (83%) were within the recommended range (Table 3). In total, 32/273 (12%) measurements were below 2 mg/L, 9/247 (4%) measurements were above 10 mg/L (recommended therapeutic index range 2–10 mg/L) and 5/26 (19%) were above 5 mg/L (recommended range 2–5 mg/L).

CV interindividual for ISZ was 54.8% and CV median intraindividual was 43.4%. In the latter, ten patients with only one value were excluded (Table 3).

#### 3.2.2. Serum Voriconazole

A total of 1331 s-VRZ measurements were performed. Of these, 89 measurements below detection level (0.2 mg/L) were excluded, leaving 1242 measurements from 283 patients (Figure 1b). The median value was 2.6 mg/L (range: 0.2–21.9 mg/L), and 827/1242 (67%) were within the recommended range of 1–5.5 mg/L (215 below and 200 above) (Table 3). Among the pediatric patients (≤16 years), the numbers of measurements within the recommended range were 61% (165/272) and 68% (662/970) among the adult patients (>16 years).

Overall, CV interindividual for s-VRZ was 83.3% whereas CV interindividual was 93% among patients ≤ 16 years and 81% among patients > 16 years. A total of 91 patients had only one measurement, leaving 192 patients for CV median intraindividual determination, which was 53.2% (Table 3).

Nine patients had more than one value of s-ISZ and more than one value of s-VRZ in the study period. Among those nine patients, CV median intraindividual was for s-ISZ 43.7% and for the same nine patients 63.3% for s-VRZ (Table 3).

### 3.3. Adverse Events

Adverse events to ISZ were reported in 33% of patients (12/36) who had s-ISZ median levels from 1.7 to 6.2 mg/L (Table 2). Abdominal malaise and elevated liver enzymes specifically were closely related to the use of ISZ in 7/36 (19%) patients, evaluated by the treating physician, but none of the adverse events led to discontinuation of the drug. All adverse events were considered as mild to moderate. Among the 12 patients with adverse events, 49% (50/102) of the measurements were above 5 mg/L. Median TDM levels for patients with and without reported adverse events are shown in Table 2.

### 3.4. Breakthrough Fungal Infection

In two patients treated for mucormycosis, microscopies of biopsies were positive with septate hyphae and culture positive for *A. fumigatus/A. flavus* and *A. fumigatus*/*A. niger*, respectively. The *Aspergillus* species were identified when TDM levels were 3.5–7 mg/L and 4.4–13.6 mg/L in the two patients, respectively.

### 3.5. Drug–Drug Interactions

**Figure 3:**
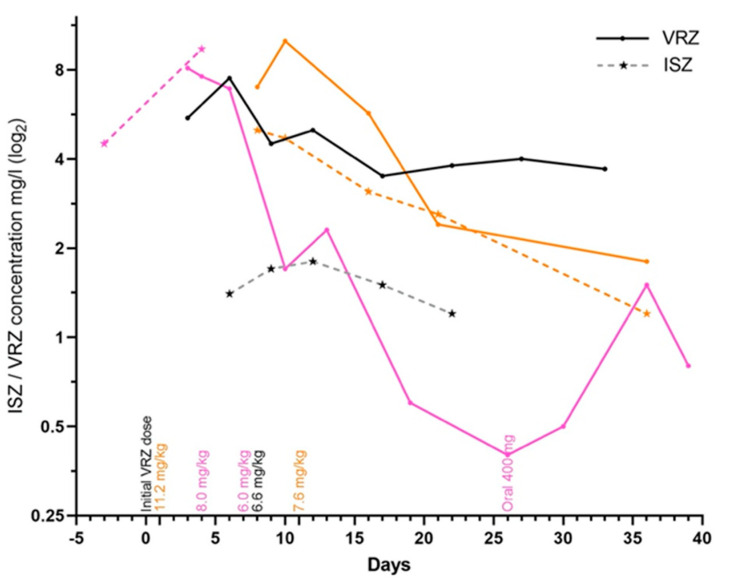
Three patient cases who during antifungal treatment switched from ISZ to VRZ. On day 0 the patients discontinued ISZ and began VRZ instead. Filled circles connected with solid lines represent serum concentrations of VRZ while asterisks connected with dashed lines represent serum concentrations of ISZ. The s-VRZ concentrations are plotted up to the first 39 days after the initial dose. The patient represented in pink received an initial VRZ dosage of 6 mg/kg twice a day. Day 4, 4 mg/kg twice a day. Day 7, 3 mg/kg twice a day. Day 26, switched to oral intake 200 mg twice a day. The patient represented in black received an initial VRZ dosage of 4.5 mg/kg twice a day. Day 8, 3.3 mg/kg twice a day. The patient represented in orange received an initial VRZ dosage of 7.5 mg/kg twice a day and had also received statins until two days after initiating ISZ. Day 1, VRZ dosage 5.6 mg/kg twice a day. Day 11, 3.8 mg/kg twice a day. None of the three patients received calcineurin inhibitors.

Three patients switched from ISZ to VRZ. One continued to have detectable s-ISZ levels in up to 35 days after suspending ISZ while treating with VRZ. The initial s-VRZ measurements were above the therapeutic range for all three patients (Figure 3). While receiving ISZ, the patients’ s-ISZ measurements were within the recommended therapeutic range (Figure 3).

Six patients received one of the immunosuppressive agents (cyclosporine, tacrolimus or sirolimus) which are known to interact with azoles (Table 1). Two patients received vincristine, of which one was dose-adjusted in vincristine to accommodate potential neurotoxicity. The other received ISZ instead of VRZ while treating with vincristine with the purpose of avoiding potential drug–drug interaction leading to neurotoxicity. S-ISZs among patients receiving cyclosporine, macrolides, statins or digoxin had TDM values from 0.5 to 6.6 mg/L.

### 3.6. Mortality

The 42-day mortality (specifically) was 6% (2/35). One patient was censored from the survival analysis with an unknown outcome (Table 1).

## 4. Discussion

The main findings in the present study were that the ISZ exposure was less variable than that of VRZ. Patients not treatable with VRZ due to side effects tolerated ISZ well. There was little clinical information regarding drug–drug interactions, but we did find that when switching from ISZ to VRZ, ISZ remained detectable up to 35 days after discontinuation and might have resulted in high initial s-VRZ measurements and potentially an extended half-life of both azoles, which are known to be 56–104 h for ISZ and only 6–12 h for VRZ [2].

The clinical phase III trial SECURE found the majority of s-ISZ measurements were within the range of 1–7 mg/L [23]. The median value in the present study was 4.3 mg/L and well within that range, the same as in other studies [7,8,9,12,17,24,25,26]. In the present study, the majority of the s-ISZ measurements were also within the recommended range, but for VRZ a third of TDM measurements were outside the recommended range, which is also in accordance with the literature [27]. However, since we had no access to clinical data for patients receiving VRZ, we excluded s-VRZ measurements below the detection level (0.2 mg/L), assuming a mistaken requisition. Therefore, it is possible we also have excluded measurements from patients actually receiving VRZ.

The median TDMs were not higher among patients who received increased ISZ dosages compared to those who did not. This might be a result of a low ISZ TDM value leading to an increase in ISZ dosing, and the fact that patients with a stabile ISZ TDM value did not need dose adjustments.

In the present study CV interindividual was not surprisingly higher than CV median intraindividual for both ISZ and VRZ. A similar tendency has been reported for posaconazole [28]. When comparing CV median intraindividual for ISZ and VRZ in the same patients who had received both VRZ and ISZ in sequence, the CV median intraindividual was lower for ISZ than VRZ. This may support that ISZ is more stable in the serum than VRZ, although the results may be biased in those patients who received ISZ prior to VRZ. TDM values from the SECURE trial found a mean CV intraindividual of 23.2% [23], which is lower than the present study’s CV median intraindividual. Other studies have reported CVs of 36.6% (interindividual) and 28.2% (median intraindividual) on maintenance dosages not exceeding 200 mg [17]. In the CPA population, mean CV intraindividual was 30% at a dosage of 100 mg and 20% at a dosage of 200 mg, suggesting that ISZ 100 mg might be adequate for CPA [14]. Others have reported ISZ CVs from 61.52% to 66% [24,26]. One of the studies found a higher ISZ CV in patients < 18 years [26], as we also did for VRZ in the pediatric group in the present study.

Adverse events to ISZ in the present study were mostly gastroenteric and hepatic, reported by the treating physician and also reported by others [12,14,25,29,30,31]. Adverse events were not closely related to high median TDMs. Adverse events in relation to the TDM result were difficult to evaluate in the present study due to other morbidity and other drug administrations that could also explain the reported adverse events as most likely.

Studies comparing adverse events to ISZ with VRZ have found higher rates of adverse events in patients treated with VRZ than ISZ [5,11,13]. Our data also support more tolerable side effects of ISZ compared to VRZ since patients treated with ISZ due to intolerance to VRZ did not discontinue ISZ.

Two patients had breakthrough fungal infections even with high levels of s-ISZ. Breakthrough fungal infections during ISZ prophylaxis or treatment have also been reported by others [7,8,9,10,11,32,33]. Breakthrough fungal infections during ISZ prophylaxis in hematological patients when s-ISZ was within the therapeutic range have also been observed [9]. This observation might be supported by the post hoc analysis of the SECURE trial, which found that VRZ seemed to have a better success rate among patients with unresolved neutropenia in the treatment of aspergillosis [34].

Our data do not support severe drug–drug interactions besides the long metabolism of ISZ when switching from ISZ to VRZ. According to the literature, ISZ has been shown to increase the level of cyclosporine, tacrolimus and sirolimus [35,36], and discontinuation of ISZ due to suspected drug–drug interaction with tacrolimus has also been reported [8]. Information regarding potentiation of immunosuppressive agent was sparse in the present study, but we do recommend monitoring the levels of calcineurin inhibitors during ISZ treatment.

The 42-day mortality was 6% in the present study. Other studies have reported 42-day mortality rates from 7.7% to 58.3%, including mortality in breakthrough fungal infections [4,5,9,30,33,37]. We ascribe the low 42-day mortality rate in the present study to the inclusion of patients who received ISZ as an empirical treatment and the inclusion of patients who had survived long enough to have switched from VRZ to ISZ.

Limitations to this study are the retrospective design and the limited number of patients. Since many studies, including ours, are retrospective [12,17,24,26], there is no certainty that the included samples were trough samples. One prospective trial did however manage to include trough levels only and found a median s-ISZ of 2.35 mg/L [25]. However, because of the long half-life of ISZ the variation between C_max_ and C_min_ is marginal [38]. Hence, this is likely a bigger limitation for VRZ having a much shorter half-life, which may also explain its larger CV values. The TDM might also in the present study have been requested too early in the treatment regimen before steady state serum levels were achieved. Other studies have reported the same limitation, but with the reasoning that the long half-life should permit a random sampling time [14]. Furthermore, we have inadequate clinical information regarding drug–drug interactions. We have no certainty of the fact that another drug’s effect was not potentiated since not all drugs are possible to monitor.

## 5. Conclusions

Isavuconazole is easier to target than VRZ and serum concentrations vary less for ISZ than VRZ. ISZ can be a good alternative to VRZ in those patients who are intolerable to VRZ. Although our data do not support substantial drug–drug interactions, we propose performing TDM after either adding or suspending other drugs, which are also metabolized through CYP3A4, and when switching from ISZ to VRZ.

## Figures and Tables

**Table 1:** Demography and clinical data of patients who received ISZ.

Male/Female	17/19
Median age and range (years)	60 (7–80)
Median weight and range (kg)	68 (24–97)
Median length and range of antifungal treatment in observation period and range (days) *	49 (5–584)
TDM level median and range (mg/L)	4.3 (0.5–15.4)
Time from ISZ initiation to first measurement median and range (days) **	7 (0–34)
First TDM measurement median and range (mg/L) **	3 (0.6–6.7)
**Underlying disease (*n*)**	
Hematological disease	16
Pulmonary disorder	13
Infectious diseases	4
Solid organ transplant	2
Other	1
**Indication for ISZ treatment (*n*)**	
Aspergillosis *** (2 proven, 3 probably, 0 possible, 15 unclassifiable)	20
Mucormycosis (5 proven)	5
Empirical (0 proven, 0 probable, 1 possible, 5 unclassifiable)	6
Empirical until identification of fungal infection for which VRZ was preferred (1 proven aspergillosis, 1 probably aspergillosis and 1 proven other mold infection)	3
Hyphae identified, but no species identification	1
Other fungal infection	1
**Patients (*n*) receiving co-medication with potential drug–drug interactions**	
Vincristine	2
Calcineurin inhibitors	5
Macrolides	4
Statins	3
Other CYP3A4 interacting drugs	3
**Outcome at day 42 (*n*)**	
Alive	33
Dead	2
Unknown	1

* Lengths of treatment are within the study period and based on 32 patients including two patients who had ISZ suspended in time intervals; ** Based on available data from 32 patients; *** Two patients had also cultured Mucorales species from airway samples, but no proven mucormycosis. Patients may be repeated under drug–drug interactions if more than one.

**Table 2:** TDM values and tolerance data.

TDM and Dosages	
Patients/samples who received increased dosages incl. patients with Mucorales (*n*) *	12/199
-TDM level total samples. Median and range (mg/L)	4.3 (0.6–15.4)
**Patients/samples who received increased dosages excl. Mucorales patients (*n*)**	7/72
-TDM level total samples. Median and range (mg/L)	3.6 (0.6–10)
**Patients/samples who did not receive increased dosages (*n*)**	22/73
-TDM level total samples. Median and range (mg/L)	4.6 (0.5–13.5)
**Prior VRZ treatment (*n*)**	15/36
Shifted to ISZ due to intolerance to VRZ	9/15
**Adverse events reported due to ISZ**	
Abdominal malaise/nausea/emesis (*n*)	5
Constipation (*n*) **	2
Elevated liver enzymes (*n*)	3
Fatigue (*n*)	2
Palpitations (*n*)	1
Neuropathy (*n*) **	1
Leg pain (*n*) **	1
**Patients with adverse events (*n*)**	12
-TDM level total samples. Median and range (mg/L)	5 (0.6–15.4)
-Samples > 5 mg/L (*n*)	50/102
**Patients with adverse events closely related to the use of ISZ (*n*)**	7
-TDM level total samples. Median and range (mg/L)	3.6 (1.7–7.8)
-Samples > 5 mg/L (*n*)	2/22
Patients without adverse events (*n*)	23
**-TDM level total samples. Median and range (mg/L)**	4.1 (0.5–13.5)
-Samples > 5 mg/L (*n*)	55/171

* One patient with Mucorales received increased dosage after the last TDM measurement. ** These patients also received vincristine; one patient’s dosage could not be retrieved. Patients may be repeated under adverse events if there are more than one.

**Table 3:** Comparison of ISZ and VRZ. Clinical data and coefficient of variation.

**Clinical Data and TDM**	**ISZ**	**VRZ**
Median age and range (years)	60 (7–80)	65 (0–86)
Patients ≤ 16 years	6% (2/36)	7% (21/283)
TDM median and range (mg/L)	4.3 (0.5–15.4)	2.6 (0.2–21.9)
Successful target rate of measurements	83% (227/273)	67% (827/1242)
**Coefficient of variation (CV%)**	**ISZ**	**VRZ**
**CV-interindividual**	54.8%	83.3%
Patients (*n*) *	35	283
**CV median intraindividual**	43.4%	53.2%
Patients (*n*)	25	192
**CV median intraindividual**	43.7%	63.3%
Patients (*n*) (Same patients who received ISZ and VRZ during the study period)	9	9

* One patient’s serum-concentrations for ISZ were performed at another laboratory and are not included in the calculations.

## Data Availability

The study data are at the possession of the authors until 30 November 2022. Data are only available after permission by the local authorities.

## Supplementary Materials

The following are available online at https://www.mdpi.com/article/10.3390/antibiotics10050487/s1, Figure S1: Dose-response curves.
