# Supplementary material for: Therapeutic Drug Monitoring of Isavuconazole: Serum Concentration Variability and Success Rates for Reaching Target in Comparison with Voriconazole
Source: Antibiotics (Basel). 2021 Apr 23;10(5):487. doi: 10.3390/antibiotics10050487 (PMC8145181; doi:10.3390/antibiotics10050487)
Supplement: Supplementary file 1 [file antibiotics-10-00487-s001.zip › antibiotics-1171385-supplementary.pdf]

antibiotics

Supplementary Material

Therapeutic Drug Monitoring of Isavuconazole: Serum Concentration Variability and Success Rates for Reaching Target in Comparison with Voriconazole

Malene Risum [1,2], Mai-Britt Vestergaard [3], Ulla Møller Weinreich [3,4], Marie Helleberg [5], Nadja Hawwa Vissing [6] and René Jørgensen [1,*]

1 Unit of Mycology, Department of Bacteria, Parasites and Fungi, Statens Serum Institut, Artillerivej 5, 2300 Copenhagen, Denmark; mlri@ssi.dk
2 Department of Haematology, Copenhagen University Hospital, Rigshospitalet, 2100 Copenhagen, Denmark
3 Department of Respiratory Diseases, Aalborg University Hospital, 9000 Aalborg, Denmark; maives@rn.dk (M.-B.V.); ulw@rn.dk (U.M.W.)
4 The Clinical Institute, Aalborg University, 9000 Aalborg, Denmark
5 Department of Infectious Diseases, Copenhagen University Hospital, Rigshospitalet, 2100 Copenhagen, Denmark; Marie.Helleberg@regionh.dk
6 Department of Paediatrics and Adolescent Medicine, Copenhagen University Hospital, Rigshospitalet, 2100 Copenhagen, Denmark; nadja.hawwa.vissing@regionh.dk
* Correspondence: renj@ssi.dk

Supplementary figure-S1

A

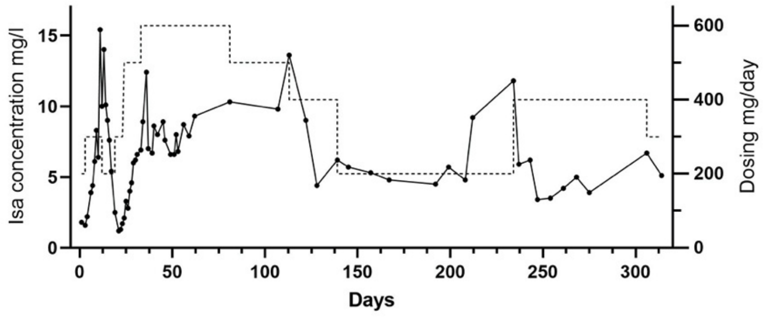

B

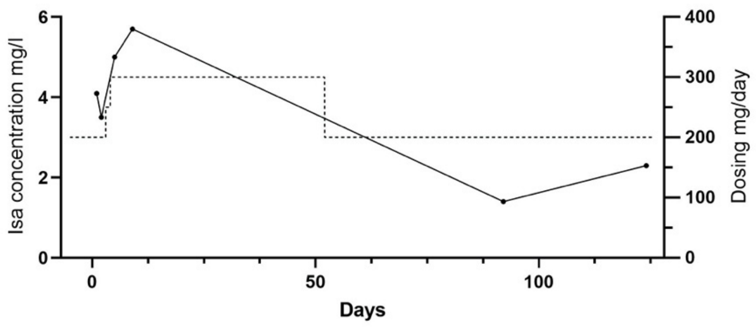

C

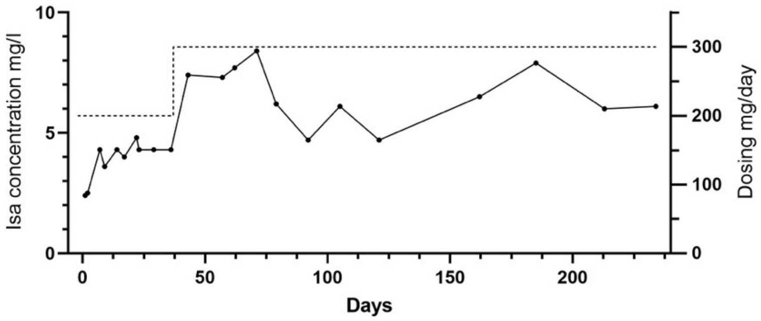

D

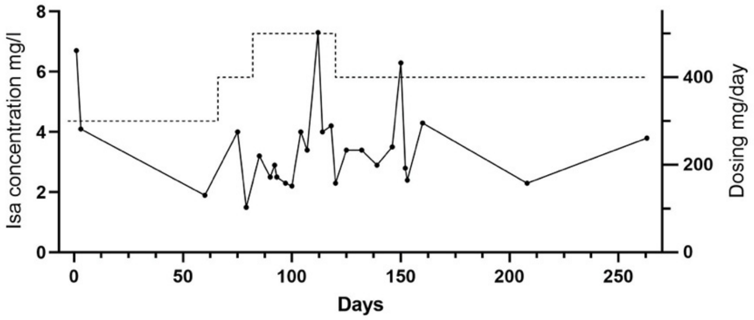

Supplementary figure S1. Dose-response curves. Patients with Mucorales receiving varying dosing throughout the treatment period. One patient with Mucorales was not adjusted in ISZ dosing during the TDM measurements and is therefore not shown. Solid line represents ISZ concentration and dashed line shows the dosing adjustments.
